# Effect of Epidural Anaesthesia on Postoperative Neutrophil-to-Lymphocyte Ratio in Paediatric Cardiac Surgery: A Retrospective Cohort Study

**DOI:** 10.7759/cureus.97964

**Published:** 2025-11-27

**Authors:** Ajay Kumar, Mayank Priyadarshi, Pooja Chandran, Sanjay Agrawal, Anish Gupta, Deepak Nehra

**Affiliations:** 1 Anesthesia, All India Institute of Medical Sciences, Rishikesh, Rishikesh, IND; 2 Neonatology, All India Institute of Medical Sciences, Rishikesh, Rishikesh, IND; 3 Cardiothoracic and Vascular Surgery, All India Institute of Medical Sciences, Rishikesh, Rishikesh, IND

**Keywords:** cardiopulmonary bypass, congenital heart surgery, epidural anesthesia, eracs, fast tracking, neuraxial anesthesia, neutrophil-lymphocyte ratio

## Abstract

Background: Paediatric cardiac surgery patients are prone to a systemic inflammatory response on cardiopulmonary bypass. Regional and epidural anaesthesia suppresses an unbalanced hyperinflammatory response generated due to the interplay within the neuro-endocrine-immune network. Regional and epidural anaesthesia reduces the inflammatory response. This study evaluated the effect of thoracic epidural anaesthesia (TEA) on the neutrophil-to-lymphocyte ratio (NLR), a marker for inflammation.

Method: This was a retrospective, observational cohort study of paediatric cardiac surgery patients conducted at a tertiary care hospital between January 2024 and June 2024. A total of 52 paediatric patients aged 1-12 years were included. The patients were categorized into TEA with general anaesthesia (TEA-GA) and isolated general anaesthesia (GA) groups. Patients in the TEA-GA group (n = 26) were compared to the isolated GA group (n = 26) for the primary outcome of NLR at 24 hours postoperatively. The secondary outcomes of the study were extubation within six hours, ventilation duration, ICU stay, postoperative stay in the hospital (PLoS), time to initiate oral intake, and arterial blood gas (ABG) parameters at the time of extubation.

Measurements and main results: Baseline characteristics, including age, gender, Society of Thoracic Surgeons-European Association for Cardio-Thoracic Surgery (STAT) score, and bypass time, were comparable between the groups. Although there was a 10-fold rise in NLR from baseline to 24-hour postoperative time, there was no significant difference in NLR at 24 hours (TEA-GA group: median = 10.75, IQR = 9.85-11.8 vs. GA group: median = 7.37, IQR = 4.45-13.95; p = 0.56). There were no significant differences in the secondary outcomes, except partial pressure of arterial carbon dioxide (31.59 ± 4.05 in the TEA-GA group vs. 34.0 ± 4.5 in the GA group; p < 0.05).

Conclusion: According to this study, TEA-GA may not affect postoperative NLR compared to GA alone in paediatric cardiopulmonary bypass.

## Introduction

The Enhanced Recovery After Surgery (ERAS) Cardiac Society has recommended a multimodal approach to reduce reliance on opioid-bound analgesia and perioperative pain management [1]. Regional blocks and thoracic epidural anaesthesia combined with general anaesthesia (TEA-GA) are part of a multimodal approach for fast-tracking paediatric cardiac surgery [1,2].

Cardiac surgery using cardiopulmonary bypass (CPB) triggers the neuro-endocrine-immune network, leading to an unbalanced hyperinflammatory response [3]. CPB leads to a widespread systemic inflammatory response that manifests as a differential leukocyte response. On one hand, stem cells increase neutrophil formation due to growth factors, and on the other hand, redistribution of lymphocytes to lymphatic organs occurs under the influence of catecholamines and cortisol. The combination of neutrophilia and lymphocytopenia, expressed as the neutrophil-to-lymphocyte ratio (NLR), holds prognostic significance as a marker of inflammation [4]. High NLR is associated with increased ICU stay, increased duration of ventilation, high inotropic score, low cardiac output, acute kidney injury, and mortality in paediatric cardiac surgical patients [4].

Erector spinae plane block (ESPB), a regional anaesthesia technique, has been reported to attenuate NLR for up to three days after cardiac surgery in adults [5]. Thoracic epidural and regional blocks help alleviate pain and have reported attenuation suppression of unbalanced hyperinflammatory response generated due to interplay within the neuro-endocrine-immune network of stress and inflammatory response by decreasing postoperative interleukin 6 (IL-6), tumour necrosis factor-alpha (TNF-α), and troponin I significantly in off-pump surgeries [5,6]. Paediatric patients are at increased risk of inflammatory response because of reactive pulmonary vasculature, an immature organ system, circuit volume up to 300% greater than the patient’s circulating volume, and higher pump flow. Any anti-inflammatory intervention has a high likelihood that its effect will manifest more in paediatric cardiac surgery patients. No literature exists regarding the impact of epidural anaesthesia on NLR in paediatric cardiac surgery.

Therefore, we evaluated the effect of thoracic epidural anaesthesia (TEA) on NLR in paediatric cardiac surgery. We hypothesized that TEA-GA would attenuate the inflammatory response following paediatric cardiac surgery compared to anaesthesia care with general anaesthesia (GA) alone. The secondary outcomes of the study were extubation within six hours, ventilation duration, ICU stay, postoperative stay in the hospital (PLoS), time to initiate oral intake, and arterial blood gas (ABG) parameters at the time of extubation.

## Materials and methods

**Figure 1:**
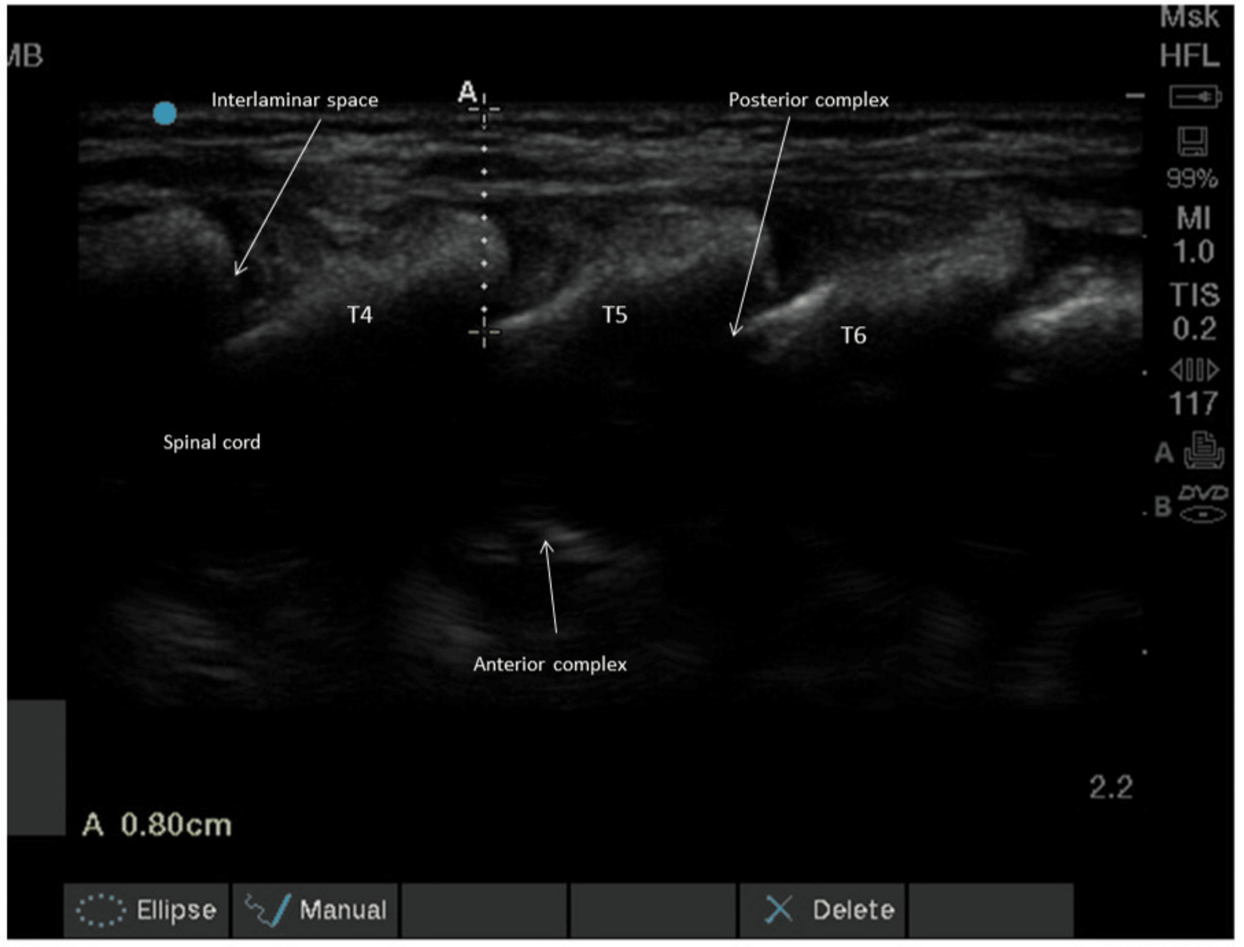
Paramedian sagittal oblique view of the midthoracic spine, anterior complex, interlaminar space, posterior complex, safety depth, and spinal cord seen as a hypoechoic shadow.

This retrospective cohort study was conducted for six months, from January 2024 to June 2024, after obtaining approval from the Institutional Ethics Committee (AIIMS/IEC/25/181). The committee exempted the requirement for individual consent. The calculated sample size, based on detecting a mean difference of at least 2 in NLR between GA and TEA groups, was 208 [5]. However, due to the time-bound nature of the study conducted over a six-month period, only 52 eligible patients could be included in the study. Patients aged between one and 12 years who underwent elective cardiac surgeries, classified as the Society of Thoracic Surgeons-European Association for Cardio-Thoracic Surgery (STAT) mortality risk of 3 or less, and American Society of Anesthesiologists (ASA) grade II and III, were included [7,8]. Patients who were excluded had a STAT score of ≥4, underwent emergency surgery, or did not require CPB. Inhalational induction was the standard practice. However, we used intravenous anaesthetic agents for patients who already had pre-existing intravenous lines. The remaining patients were induced with sevoflurane, atracurium at 0.5 mg/kg, and fentanyl at a bolus dose of 2 µg/kg. The anaesthetist intubated patients using appropriately sized endotracheal tubes after achieving adequate muscle relaxation. Haemodynamic monitoring and vasopressor infusion were managed using femoral arterial catheterization and internal jugular venous cannulation, respectively. Standard ASA-recommended monitors were also applied. All patients received intravenous methylprednisolone at a dose of 30 mg/kg prior to the initiation of CPB. Patients were divided into two groups based on anaesthesia protocols: those who received thoracic epidural anaesthesia in combination with general anaesthesia (TEA-GA), and those who received general anaesthesia (GA) alone without any nerve block. In the TEA-GA group, three spinous processes from T4 to T6 were identified and marked. The depth of the epidural space was measured using a linear ultrasound probe in a parasagittal oblique view to estimate the needle depth (Figure 1).

We used 18G epidural needles for patients weighing more than 10 kg and 20G epidural needles (Perifix One Pedia, B. Braun Medical Inc., Bethlehem, PA) for those weighing less than 10 kg. The epidural needle was advanced using the loss-of-resistance technique near the estimated depth. Epidural medications consisted of 1 mL/kg of 0.25% bupivacaine combined with 50 µg/kg of preservative-free morphine at the time of induction, followed by a maintenance infusion of 0.125% bupivacaine at 0.1 mL/kg/h. Sevoflurane and atracurium infusion at 4 µg/kg/h were used for maintenance of anaesthesia without additional opioids. A one-hour interval was ensured between epidural catheter placement and heparin administration. The epidural catheter was removed 48 hours postoperatively, and patients were monitored for up to 72 hours for any signs of motor weakness suggestive of epidural haematoma. In the GA-only group, anaesthesia was induced without prior epidural block. Fentanyl infusion was started at 0.5 µg/kg/h and continued in the ICU until extubation. Dexmedetomidine infusion was initiated at 0.25 µg/kg/h during weaning from bypass and continued up to 12 hours post-extubation. We gave an equivalent dosage of opioid, considering 1 mcg of IV fentanyl = 10 mcg epidural morphine [9]. For patients requiring mechanical ventilation beyond six hours, continuous infusions of fentanyl (0.5 µg/kg/h) and dexmedetomidine and atracurium as a muscle relaxant titrated with haemodynamics and sedation were maintained.

Blood samples for haemogram were collected preoperatively and at 12, 24, 48, and 72 hours, as well as on postoperative day five, following institutional protocol. Clinical data were retrieved from the hospital’s electronic medical record system (e-Hospital software).

We compared both groups (TEA-GA vs. GA) for the primary outcome, i.e., NLR at the 24-hour postoperative mark. The 24-hour postoperative period is the time when the NLR ratio is at its peak, as there is a pinnacle of neutrophil surge as well as a trough lymphocyte level due to ingress into the lymphoid system. Secondary outcomes included extubation within six hours postoperatively, duration of mechanical ventilation, ICU stay, PLoS, and time to initiation of oral intake after extubation. ABG parameters, such as pH, arterial oxygen to inspired oxygen ratio (PaO₂/FiO₂), and arterial carbon dioxide (PaCO₂) levels at the time of extubation, were also compared.

Statistical analysis

We performed statistical analysis using IBM SPSS Statistics for Windows, version 20.0 (IBM Corp., Armonk, NY). Group comparisons for continuous variables were conducted using the independent samples t-test, while non-parametric data were analysed using the Mann-Whitney U test. Categorical variables were compared using the chi-square test or Fisher's exact test, as appropriate. To account for repeated measurements within subjects, a linear mixed-effects model was used to assess time-specific trends in NLR between the two groups. A post-hoc analysis was done to check the power of the study. A p-value < 0.05 was considered statistically significant.

## Results

**Figure 2:**
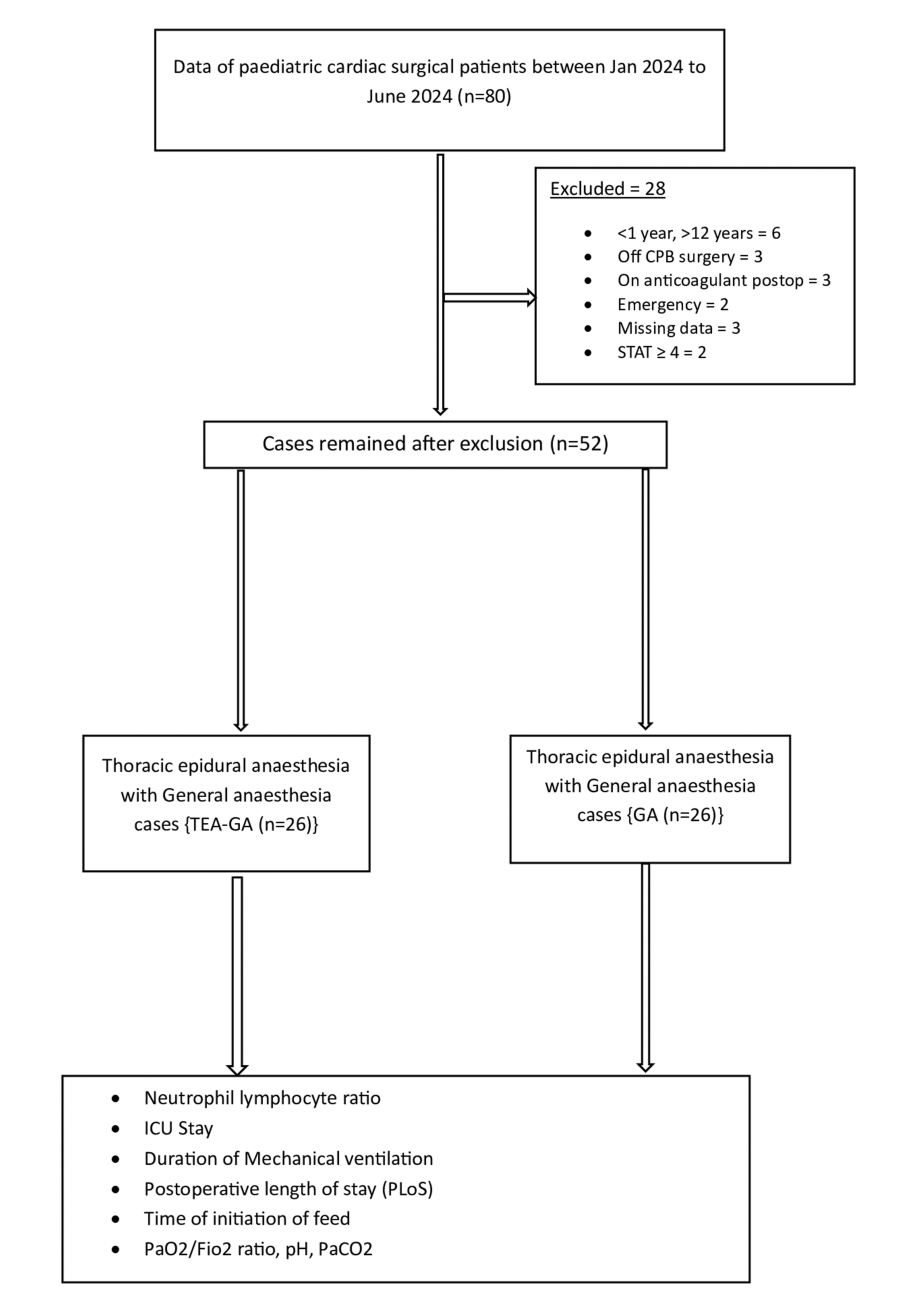
Study flow diagram. CPB: cardiopulmonary bypass; STAT: Society of Thoracic Surgeons-European Association for Cardio-Thoracic Surgery; PaO₂/FiO₂ ratio: arterial oxygen to inspired oxygen ratio; PaCO₂: partial pressure of carbon dioxide.

A total of 80 paediatric cardiac surgery patients underwent surgery between January 2024 and June 2024. After excluding 18 patients for various reasons (Figure 2), 52 patients were included in the study.

Of these, 26 patients received TEA-GA, and 26 received GA alone. Demographic characteristics and baseline variables, including age, body surface area (BSA), STAT scores, CPB time, and surgical duration, were comparable between the two groups (Table 1).

**Table 1:** Demographic characteristics and baseline haematological parameters. Data are presented as mean ± SD, median (IQR), or n (%) as appropriate. P-values derived using the Mann–Whitney U test for non-parametric continuous variables. † P-values derived using an independent samples t-test for normally distributed continuous variables. ‡ P-values derived using the chi-square test for categorical variables with sufficient counts. § P-values derived using Fisher’s exact test for sparse categorical data. A p-value < 0.05 was considered statistically significant. Statistical significance was set at p < 0.05. ALC: absolute lymphocyte count; ANC: absolute neutrophil count; ASA: American Society of Anesthesiologists; BSA: body surface area; CHD: congenital heart disease; CPB: cardiopulmonary bypass time; GA: general anaesthesia; ICU days: duration of ICU stay; MV: duration of mechanical ventilation; NLR: neutrophil-to-lymphocyte ratio; Overall: total cohort including two groups; STAT: Society of Thoracic Surgeons-European Association for Cardio-Thoracic Surgery; TEA-GA: thoracic epidural anaesthesia with general anaesthesia; TLC: total leukocyte count.

Baseline parameter	Overall (n = 52)	Group TEA-GA (n = 26)	Group GA (n = 26)	Test statistic	p-value
Age (years), Median (IQR)	5.5 (3-8)	5 (3-8)	6 (3-8)	U = 329.0	0.94^*^
Weight (kg), Median (IQR)	14 (11-20.5)	14 (11-23)	15 (11-19)	U = 330.5	0.97^*^
BSA (m^2^), Mean ± SD	0.719 ± 0.30	0.75 ± 0.33	0.68 ± 0.26	t(50)=0.83	0.41^†^
Gender, n (%)				Fisher’s exact	0.40^§^
Male	30 (57.7%)	13 (50%)	17 (65.4%)		
Female	22 (42.3%)	13 (50%)	9 (34.6%)		
Type of CHD, n (%)				Fisher’s exact	0.25^§^
Acyanotic (CHD)	33 (63.5%)	19 (73.1%)	14 (53.8%)		
Cyanotic (CHD)	19 (36.5%)	7 (26.9%)	12 (46.2%)		
STAT, n (%)				Fisher’s exact	1.00^§^
1	42 (80.7%)	21 (80.7%)	21 (80.7%)		
2	8 (15.4%)	4 (15.4%)	4 (15.4%)		
3	2 (3.9%)	1 (3.9%)	1 (3.9%)		
ASA physical status, n (%)				Fisher’s exact	0.07^§^
II	16 (31%)	11 (42%)	5 (19%)		
III	36 (69%)	15 (58%)	21 (81%)		
CPB time (min), Mean ± SD	93.4 ± 40.4	90.9 ± 42.1	95.9 ± 39.3	t(50)=0.44	0.66†
Aortic cross-clamp (min), Median (IQR)	61.5 (36-88)	61 (36-88)	68 (43-85)	U = 325.0	0.92^*^
Surgical time (min), Mean ± SD	275 ± 77.3	272.5 ± 73.8	277.69 ± 82	t(50)=0.24	0.81†
Haematological parameters					
NLR (Baseline), Median (IQR)	0.95 (0.50-1.935)	1.124 (0.744-2.026)	0.789 (0.44-1.87)	U = 296.0	0.23^*^
TLC (×10³/µL), Mean ± SD	9.55 ± 4.49	9.43 ± 5.19	9.67 ± 3.76	t(50)=0.20	0.84^†^
ANC (×10³/µL), Median (IQR)	3.36 (2.72-4.54)	3.83 (2.83-4.51)	3.31 (2.69-4.57)	U = 317.0	0.69^*^
ALC (×10³/µL), Median (IQR)	3.75 (2.59-5.01)	3.39 (2.62-4.48)	3.99 (2.54-6.69)	U = 309.0	0.27^*^

Table 2 shows the outcomes of NLR, PaCO₂, pH, PaO₂/FiO₂ ratio at various time points, ventilation duration, ICU stay length, and PLoS.

**Table 2:** Comparison of outcome variables between the groups. Data are presented as mean ± SD, median (IQR), or n (%) as appropriate. P-values were derived using the Mann–Whitney U test for non-parametric continuous data. † P-values were derived using the unpaired Student’s t-test for normally distributed continuous data. ‡ P-values were derived using the chi-square test. § P-values were derived using Fisher’s exact test. ^ P-value derived from a mixed-effects linear regression model comparing the rate of change in NLR over time between groups. A p-value < 0.05 was considered statistically significant. Overall: total cohort including two groups; TEA-GA: thoracic epidural anaesthesia with general anaesthesia; GA: general anaesthesia; ANC: absolute neutrophil count; ALC: absolute lymphocyte count; Feed: initiation of post-extubation feed; ICU: intensive care unit; HFNC: high-flow nasal cannula; IQR: interquartile range; MV: duration of mechanical ventilation; NLR: neutrophil-to-lymphocyte ratio; PLoS: postoperative length of stay; SD: standard deviation; TLC: total leukocyte count.

Parameters	Overall (n = 52)	Group TEA-GA (n = 26)	Group GA (n = 26)	Test statistic	p-value
NLR 24 hours, Median (IQR)	8.13 (4.53-15.69)	10.75 (9.85-11.8)	7.37 (4.45-13.95)	U = 317.5	0.56^*^
NLR, Median (IQR)					
Baseline	0.95 (0.50-1.94)	1.12 (0.74-2.03)	0.79 (0.44-1.87)	U = 310.0	0.23*
12 hours	5.18 (3.6-8.8)	5.48 (3.61-9.12)	4.9 (2.96-8.0)	U = 328.0	0.58*
24 hours	8.13 (4.53-15.69)	10.75 (9.85-11.8)	7.37 (4.45-13.95)	U = 317.5	0.56*
48 hours	7.2 (3.56-10.48)	8.46 (4.7-10.6)	5.94 (3.4-9.29)	U = 298.0	0.18*
72 hours	4.0 (2.0-8.0)	3.5 (2.0-8.0)	4.0 (2.0-9.0)	U = 331.0	0.75*
Overall difference in NLR trend over time	------	-------	-------	Mixed effects model	0.81^
TLC (×10³/µL) 24 hours, Mean ± SD	15.69 ± 5.81	15.61 ± 6.81	15.76 ± 4.74	t = 0.09	0.93†
ANC (×10³/µL) 24 hours, Median (IQR)	1.15 (9.68-15.5)	1.28 (9.68-15.8)	1.13 (9.99-13.7)	U = 315.0	0.70^*^
ALC (×10³/µL) 24 hours, Median (IQR)	1.35 (0.828-2.32)	1.26 (0.828-1.72)	1.89 (9.15-24.8)	U = 308.0	0.31^*^
Extubation < 6 hours, n (%)	31 (59.6%)	18 (69.2%)	13 (50%)	Fisher’s exact test	0.40^§^
Mechanical ventilation (min), Median (IQR)	270 (180-720)	240 (180-375)	300 (210-720)	U = 303.0	0.22^*^
ICU stay (days), Mean ± SD	3.21 ± 1.38	3.0 ± 1.54	3.39 ± 1.19	t = 0.87	0.39^†^
Postoperative hospital stay (days), Mean ± SD	6.21 ± 1.79	6.125 ± 1.87	6.3 ± 1.74	t = 0.35	0.73^†^
Time to feed (h), Median (IQR)	9 (6-14)	8 (5-14)	10 (8-14)	U = 287.5	0.09^*^
HFNC use, n (%)	11 (21.2%)	5 (19.2%)	6 (23.1%)	Fisher’s exact test	1.00^§^
Extubation PaCO₂ (mmHg), Mean ± SD	32.77 ± 4.4	31.59 ± 4.05	34.0 ± 4.5	t = 2.02	0.05^†^
Mortality, n (%)	6 (11.5%)	3 (11.5%)	3 (11.5%)	Fisher’s exact test	1.00^§^

**Figure 3:**
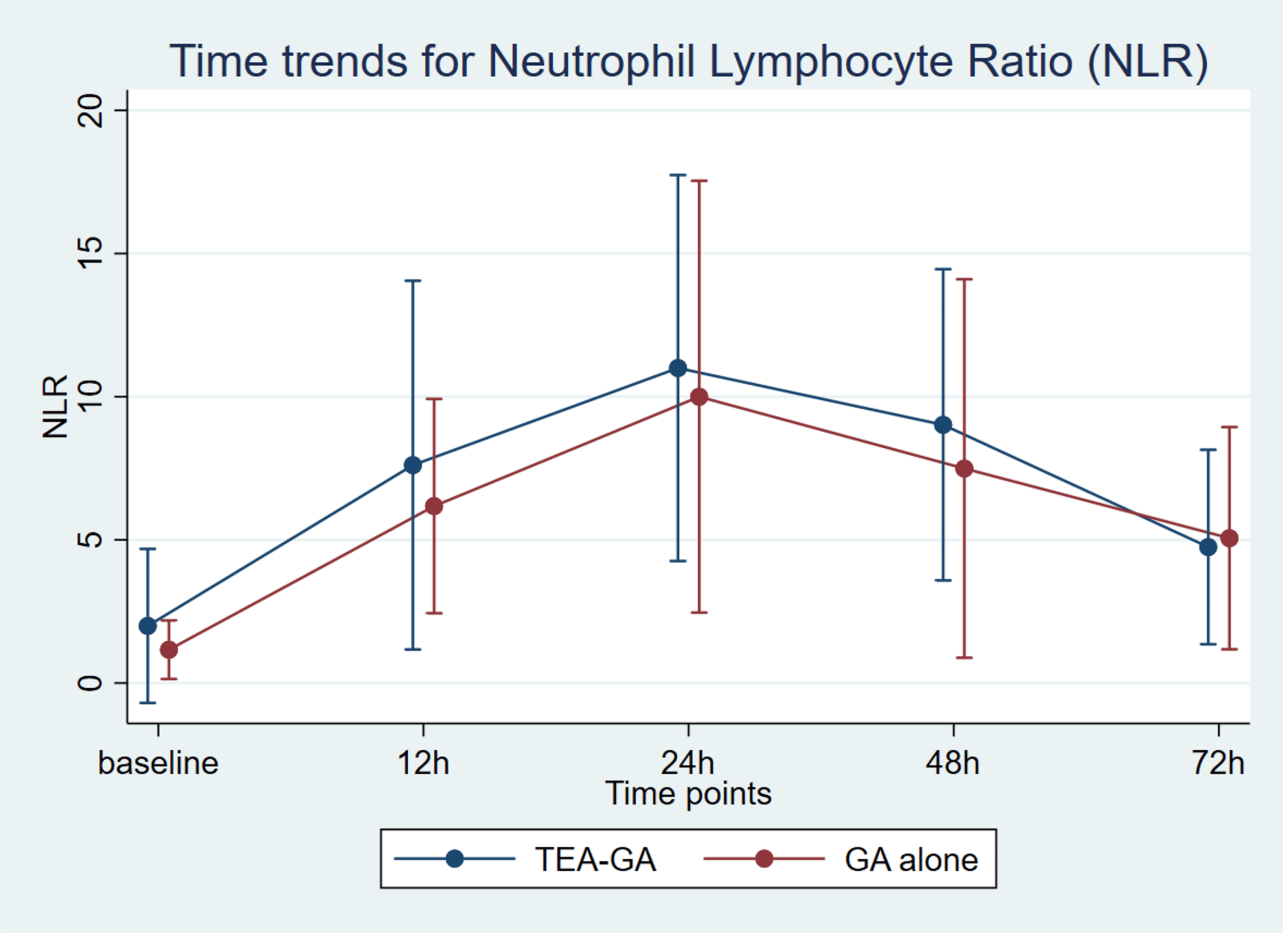
NLR trends during perioperative period upto 72 hours. TEA-GA: thoracic epidural anaesthesia with general anaesthesia; GA: general anaesthesia.

There was no significant difference in the primary outcome of NLR at 24 hours postoperatively between the two groups. The median (IQR) NLR was 10.75 (9.85-11.8) in the TEA-GA group and 7.37 (4.45-13.95) in the GA group (p = 0.56). Moreover, NLR observed at 12, 48, and 72 hours after surgery was not significantly different. Even when patients were divided into two age groups (one to six years and seven to 12 years), NLR was not significantly different between the groups at 12, 24, 48, and 72 hours, respectively. Although NLR increased significantly over time in the overall study population, no group-wise differences or group-specific time trends were observed (Figure 3).

Likewise, no significant differences were found between the groups in terms of extubation within six hours, duration of mechanical ventilation, ICU stay, PLoS, or time to feed initiation after extubation. Our study was underpowered (post-hoc power of 28%) to detect a significant difference in NLR between the groups.

PaCO₂ levels were significantly lower in the TEA-GA group (31.59 ± 4.05 mmHg) compared to the GA group (34.0 ± 4.5 mmHg; p = 0.05). Other ABG parameters, including pH, serum bicarbonate, base deficit, lactate, and the PaO₂/FiO₂ ratio, showed no significant differences between the groups.

Similarly, haematological parameters such as haemoglobin, platelet count, total leukocyte count, neutrophil count, and lymphocyte count were comparable across both groups. There were three deaths reported in each group.

## Discussion

This study evaluated the effect of TEA-GA on the postoperative NLR. The results indicated that TEA-GA had no significant impact on NLR compared to GA alone during the 24-hour postoperative period. Additionally, TEA-GA did not offer any advantage in reducing the ventilation duration, ICU stay length, PLoS, or time to initiation of oral feeding. No cases of epidural haematoma were observed in the TEA-GA group.

Lv et al. reported the anti-inflammatory effects of epidural anaesthesia, evidenced by decreased levels of IL-6 and TNF-α in patients undergoing coronary artery bypass grafting (CABG). [6] As a neuraxial block, epidural anaesthesia modulates both the local inflammatory response and central regulatory pathways, including the sympathetic nervous system and the hypothalamic-pituitary-adrenal (HPA) axis [3]. It has also been shown to reduce CRP levels in the postoperative period in paediatric cardiac surgery [2]. Aykut et al. reported a significantly smaller increase in NLR during the first two postoperative days in patients undergoing CABG under ESPB combined with GA [5].

Unlike previous studies, our research found that the NLR increased to nearly 10 times the baseline value in both groups at 24 hours postoperatively. The epidural block did not significantly affect NLR at any time point up to three days after surgery. The lack of effect of TEA-GA on the immune response may be attributed to the presence of multiple inflammatory triggers associated with CPB, which are absent in off-pump CABG procedures [10]. In non-cardiac surgeries, the immune response is primarily influenced by the local surgical site, central sympathetic activation, the HPA axis, and blood transfusions [10]. In contrast, cardiac surgeries involving CPB introduce additional inflammatory triggers such as contact activation from the extracorporeal circuit, ischaemia-reperfusion injury, and endotoxaemia [10].

Even though the epidural block may modulate the central sympathetic and HPA axis responses, this modulation may be insufficient to counteract the intense inflammatory response triggered by CPB. Additionally, both groups received equipotent doses of different opioids, albeit through different routes, which likely had minimal influence on NLR and contributed to the lack of significant difference [9].

Our findings differ from those reported by Aykut et al., potentially due to differences in the type of regional intervention used, patient age group, and underlying disease profiles [5]. Furthermore, Şahin et al. found no significant difference in NLR between patients receiving total intravenous anaesthesia and those receiving inhalational anaesthesia, supporting the notion that the anaesthesia technique, whether neuraxial, inhalational, or intravenous, may not significantly impact NLR in cardiac surgery [11].

Some studies have reported no anti-inflammatory effects of neuraxial anaesthesia on inflammatory markers in cardiac surgery patients. Ganapathy et al. compared the effect of thoracic epidural on CABG with or without CPB and found no significant difference in IL-6 levels at the end of surgery [12]. Similarly, Lee et al. observed comparable levels of IL-6 and TNF-α at all time points in patients with CABG and valvular disease who were randomly assigned to receive either high spinal anaesthesia or GA alone [13]. Furthermore, a meta-analysis evaluating the benefits and limitations of epidural anaesthesia in cardiac surgery did not address its effect on the innate or inflammatory immune response [14].

Regarding early extubation within six hours, our findings were comparable to those of a retrospective observational study that reported a 55% extubation rate in the epidural anaesthesia cohort [15]. However, our rate was lower than that observed in another cohort, where early extubation occurred in 83% of patients receiving high spinal anaesthesia and 62% of those receiving GA [16]. We attribute this variation to the younger age group in our study, which is generally more challenging to extubate. The duration of ventilation did not differ significantly between the groups, consistent with findings from other studies [17]. ICU stay and PLoS were comparable between groups, aligning with the results of a study involving caudal epidural block [18].

We utilized pre-procedural ultrasound (USG) to measure the safety depth prior to epidural placement in paediatric cardiac surgery patients. The paraspinous oblique view of thoracic vertebrae assisted in the estimation of the depth of the epidural space, thus preventing any inadvertent injury to the spinal cord (Figure 1) [19]. No complications such as epidural haematoma or motor weakness due to cord injury were reported in our study. In the paediatric population, the epidural space has relatively compliant tissue with fewer septations, which facilitates the cranial spread of drugs in the caudal epidural approach. This anatomical characteristic may partly explain the rare occurrence of epidural haematoma in children. The caudal epidural technique is widely and safely used in paediatric cardiac surgery without significant complications. However, TEA offers additional advantages, such as targeted drug delivery using smaller volumes, reduced opioid requirements, minimal impact on gastrointestinal motility, and a lower risk of infection.

Despite these advantages, the small sample size in our study limits the ability to conclusively establish the safety of TEA in paediatric cardiac surgery. Previous studies have reported an incidence of epidural haematoma ranging from one in 150,000-220,000, comparable to that in non-cardiac surgery. Nonetheless, we can take appropriate precautions to further minimize the risk of complications related to the epidural catheter [20].

This study had several limitations. It was a retrospective, observational, single-centre study, which was underpowered (post-hoc power of 28%) to detect a significant difference in NLR between the groups. The study had a limited sample size and a wide age range of participants (1-12 years). All patients were administered intravenous dexamethasone and methylprednisolone as standard practice in paediatric CPB, which might have affected the immune response. Additionally, we were unable to assess pain scores and sedation levels due to inadequate documentation. However, a prospective study with a large sample size may give a meaningful comparison.

## Conclusions

In conclusion, TEA-GA may not significantly affect postoperative NLR compared to GA alone in paediatric cardiac surgery patients undergoing CPB. Clinical outcomes, including extubation within six hours, duration of mechanical ventilation, ICU stay, postoperative length of stay, and time to initiation of oral intake, did not show any fast-tracking advantage with TEA. TEA was feasible and safe in paediatric cardiac surgery patients when placed under ultrasound guidance, with no reported complications such as epidural haematoma or motor weakness during the study period. However, a prospective study with a large sample size may give a meaningful comparison.
